# Supplementary figures and images for: Mono-methylated histones control PARP-1 in chromatin and transcription
Source: eLife. 2024 May 1;13:RP91482. doi: 10.7554/eLife.91482 (PMC11062633; doi:10.7554/eLife.91482)

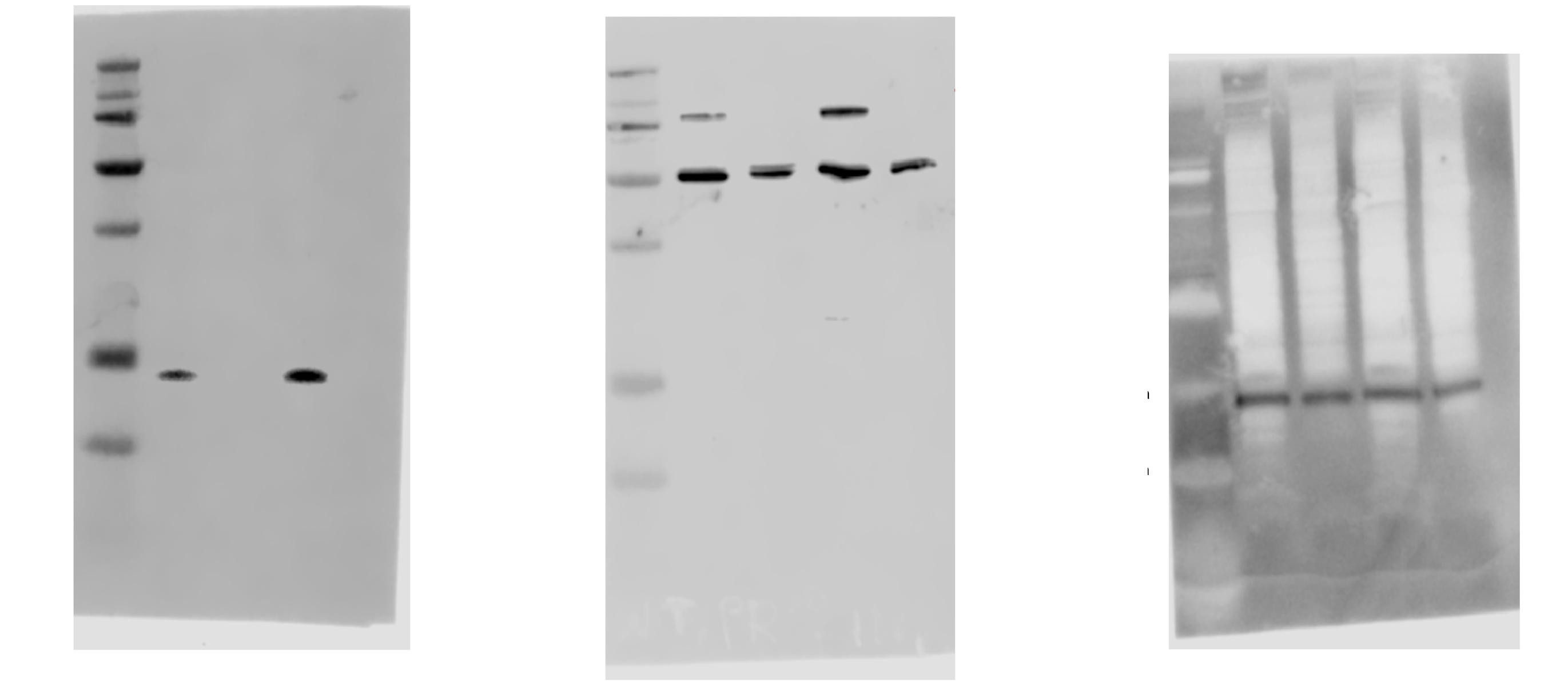

Supplement: Figure 3—figure supplement 1—source data 1. [file elife-91482-fig3-figsupp1-data1.zip › Figure 3 - figure supplement 1 - Source data 1.tif]

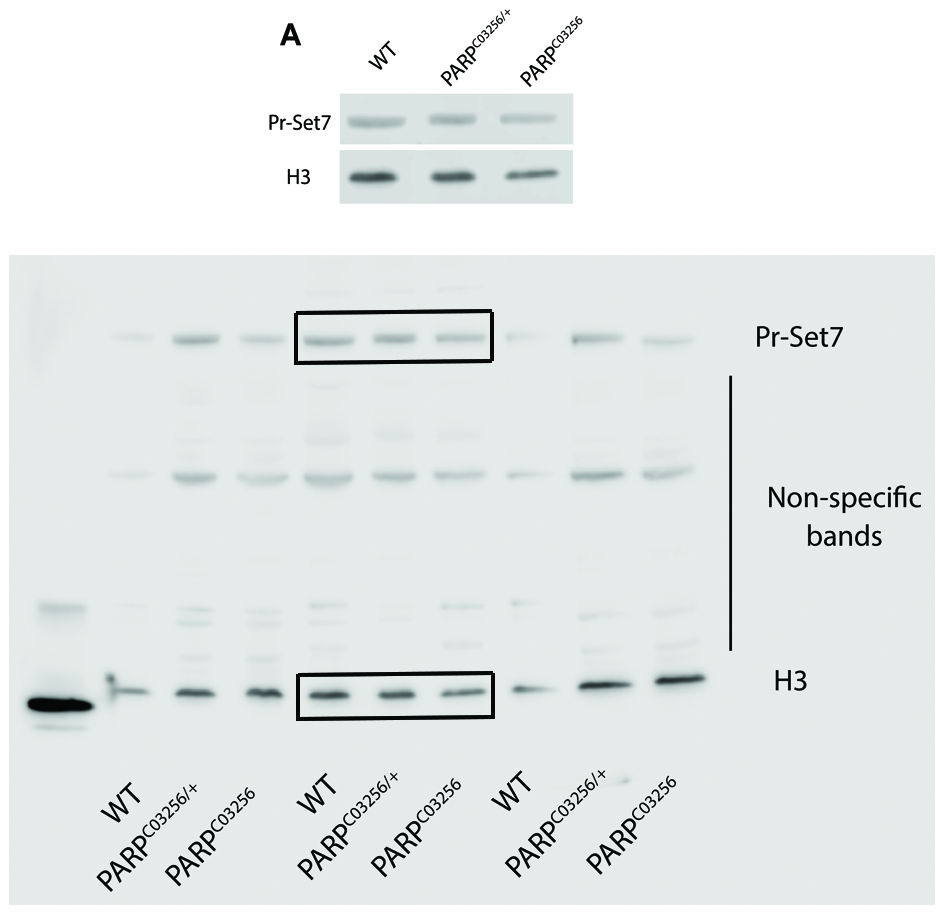

Supplement: Figure 3—figure supplement 1—source data 2. [file elife-91482-fig3-figsupp1-data2.zip › Figure 3 - figure supplement 3A - Source data 2.tif]

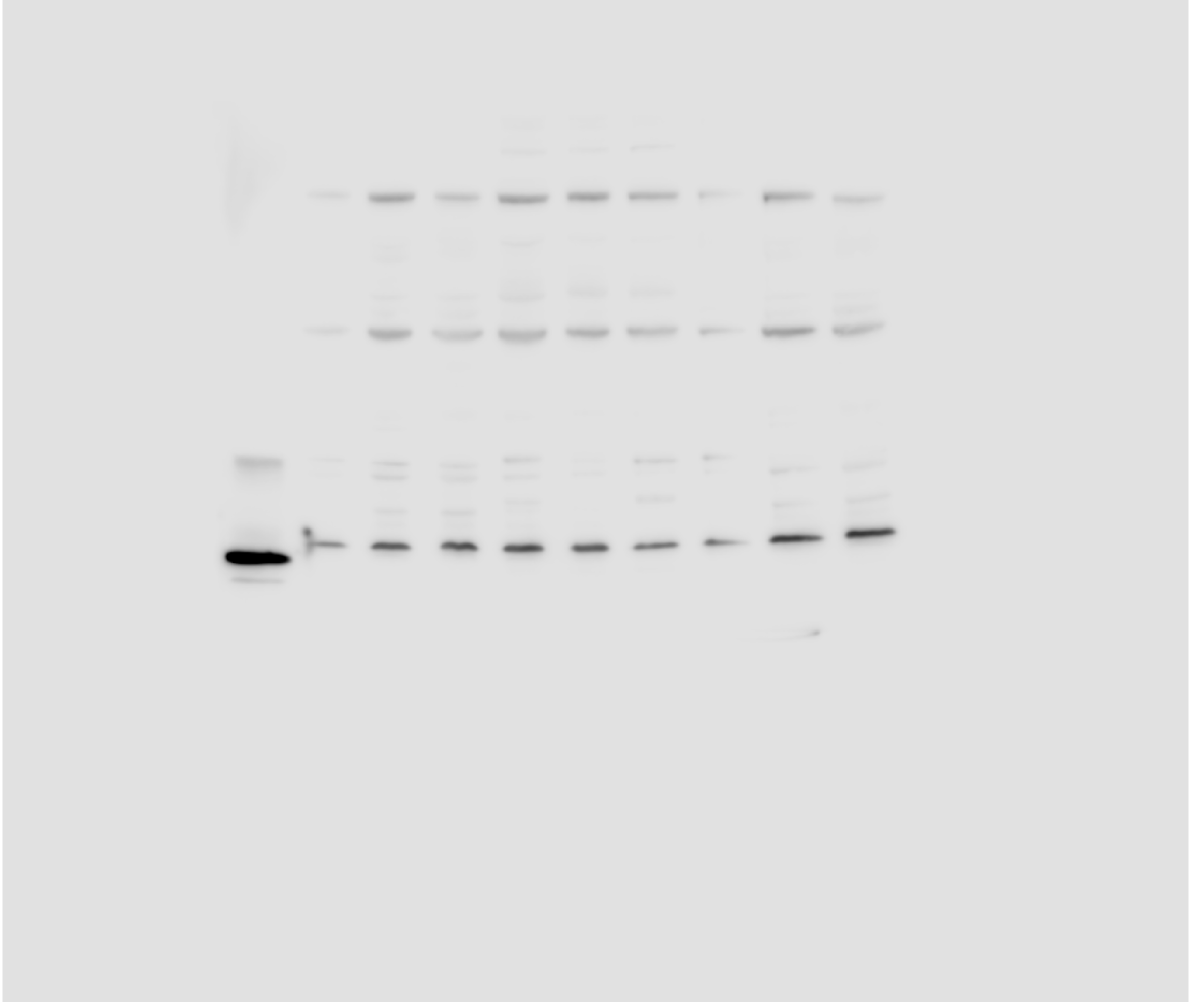

Supplement: Figure 3—figure supplement 3—source data 1. [file elife-91482-fig3-figsupp3-data1.zip › Figure 3 - figure supplement 3A - Source data 1.tif]

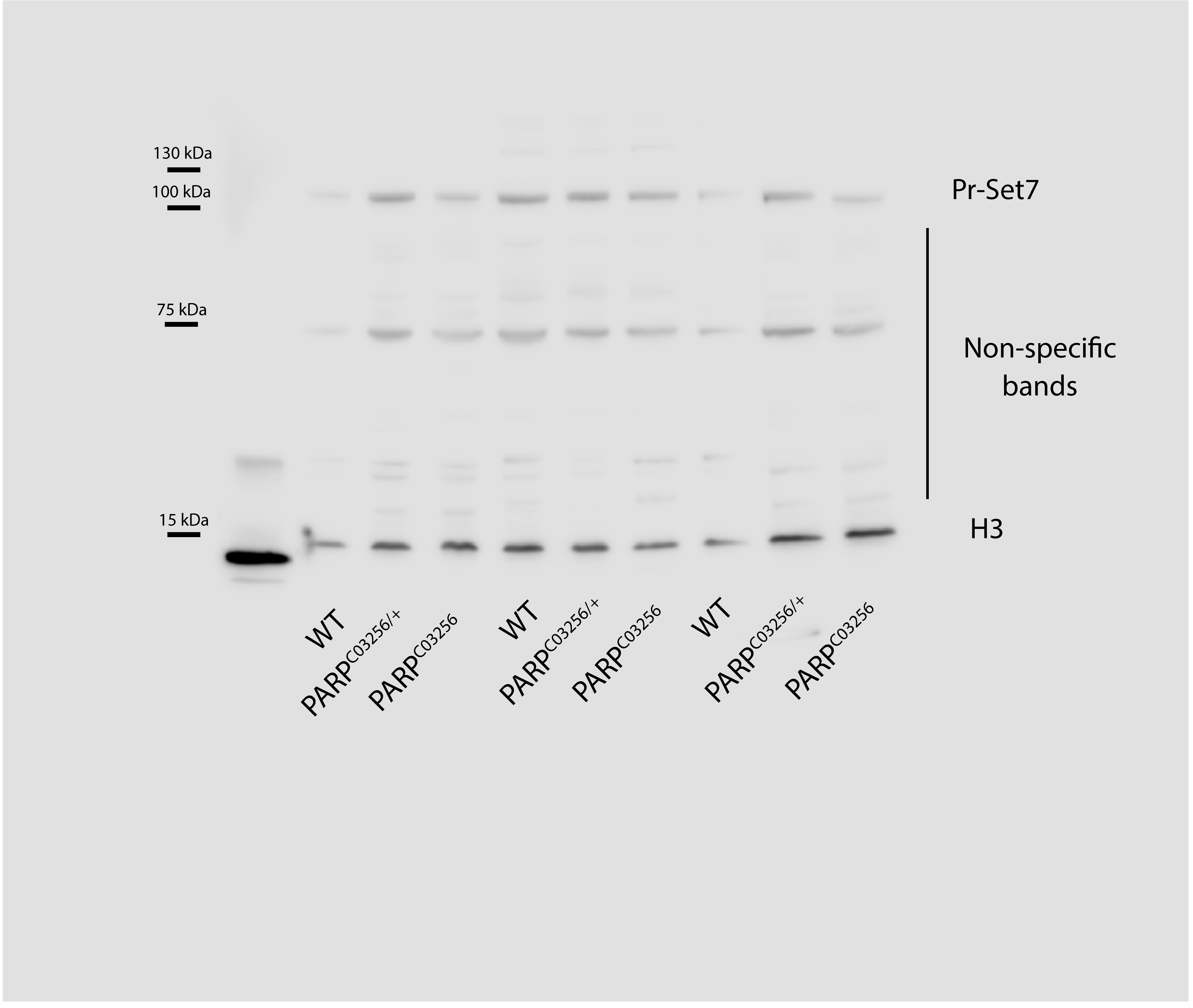

Supplement: Figure 3—figure supplement 3—source data 2. [file elife-91482-fig3-figsupp3-data2.zip › Figure 3 - figure supplement 3 - Source data 2/Figure 3 - figure supplement 3 - Source data 2.tif]

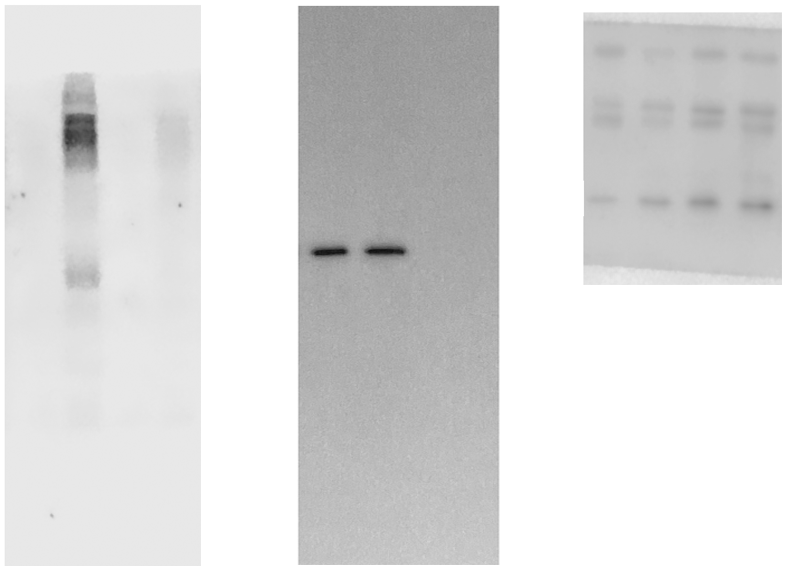

Supplement: Figure 5—source data 1. [file elife-91482-fig5-data1.zip › Figure 5B - Source data 1.tif]

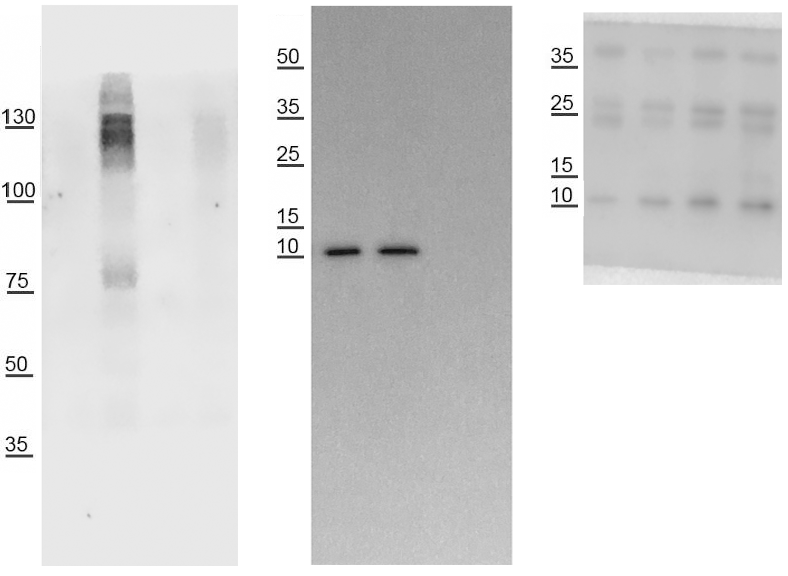

Supplement: Figure 5—source data 2. [file elife-91482-fig5-data2.zip › Figure 5B - Source data 2/Figure 5B - Source data 2.tif]
